# Supplementary material for: Developing a Microsatellite Polymerase Chain Reaction System for Small Yellow Croaker (Larimichthys polyactis) and Its Application in Parentage Assignment
Source: Biology (Basel). 2024 Sep 11;13(9):710. doi: 10.3390/biology13090710 (PMC11428518; doi:10.3390/biology13090710)
Supplement: Supplementary file 1 [file biology-13-00710-s001.zip › Supplementary materials_Table S1.pdf]

Table S1. The results of parentage analysis.

Offspring	Candidate parents 1	Pair loci		LOD score	Candidate parents 2	Pair loci		LOD score	Trio loci		LOD score	Confidence
		Compare	Mismatch			Compare	Mismatch		Compare	Mismatch		
Offs-001	Parent-14	9	0	7.48E+00	Parent-18	9	2	-1.88E+00	9	2	8.77E+00	*
Offs-002	Parent-14	9	1	6.62E-01	Parent-18	9	1	3.96E+00	9	2	7.13E+00	*
Offs-003	Parent-18	9	0	7.79E+00	Parent-28	9	2	-4.02E+00	9	2	7.43E+00	*
Offs-004	Parent-15	9	2	-6.42E+00	Parent-73	9	1	-1.54E+00	9	5	-1.28E+01	-
Offs-005	Parent-14	9	2	-1.78E+00	Parent-18	9	1	3.73E+00	9	3	3.90E+00	*
Offs-006	Parent-14	9	1	3.59E+00	Parent-18	9	0	7.83E+00	9	2	1.05E+01	*
Offs-007	Parent-14	9	1	3.75E+00	Parent-18	9	0	8.32E+00	9	1	1.57E+01	*
Offs-008	Parent-14	9	1	1.59E+00	Parent-18	9	0	8.31E+00	9	2	9.24E+00	*
Offs-009	Parent-14	9	0	8.16E+00	Parent-18	9	0	9.02E+00	9	1	1.70E+01	*
Offs-010	Parent-14	9	0	7.89E+00	Parent-18	9	1	4.38E+00	9	1	1.65E+01	*
Offs-011	Parent-06	9	2	-1.59E+00	Parent-66	9	3	-5.68E+00	9	3	3.20E+00	*
Offs-012	Parent-08	9	2	-9.17E-01	Parent-14	9	0	8.48E+00	9	2	1.08E+01	*
Offs-013	Parent-14	9	2	-3.11E+00	Parent-18	9	1	2.40E+00	9	3	1.10E+00	*
Offs-014	Parent-18	9	1	2.62E+00	Parent-28	9	1	1.16E+00	9	2	6.50E+00	*
Offs-015	Parent-18	9	1	2.88E+00	Parent-21	9	2	-4.28E+00	9	2	6.38E+00	*
Offs-016	Parent-22	9	1	6.97E+00	Parent-40	9	1	9.22E+00	9	2	1.66E+01	*
Offs-017	Parent-14	9	1	2.86E+00	Parent-18	9	3	-6.15E+00	9	3	2.81E+00	*
Offs-018	Parent-07	9	2	-3.43E+00	Parent-18	9	1	2.41E+00	9	3	2.60E-01	+
Offs-019	Parent-08	9	2	-2.59E+00	Parent-14	9	1	5.08E+00	9	3	4.45E+00	*
Offs-020	Parent-14	9	2	-2.59E+00	Parent-33	9	1	1.23E-01	9	3	1.84E-01	+
Offs-021	Parent-14	9	1	2.70E+00	Parent-18	9	0	7.59E+00	9	2	9.55E+00	*
Offs-022	Parent-18	9	1	2.28E+00	Parent-21	9	2	-4.74E+00	9	2	3.79E+00	*
Offs-023	Parent-18	9	0	9.34E+00	Parent-83	9	2	-3.02E+00	9	2	8.89E+00	+
Offs-024	Parent-14	9	2	-2.19E-01	Parent-18	9	1	7.57E-01	9	2	6.41E+00	*
Offs-025	Parent-14	9	1	5.16E+00	Parent-18	9	1	3.77E-01	9	2	8.10E+00	*

Offs-026	Parent-73	9	0	3.94E+00	Parent-80	9	2	-1.73E+00	9	3	1.58E+00	*
Offs-027	Parent-73	9	0	5.01E+00	Parent-80	9	2	-1.88E+00	9	3	2.29E+00	*
Offs-028	Parent-14	9	1	2.18E+00	Parent-18	9	0	8.84E+00	9	3	5.54E+00	*
Offs-029	Parent-14	9	1	2.81E+00	Parent-18	9	0	7.74E+00	9	3	5.43E+00	*
Offs-030	Parent-14	9	1	5.04E+00	Parent-18	9	0	9.16E+00	9	1	1.72E+01	*
Offs-031	Parent-14	9	1	1.20E+00	Parent-18	9	1	2.28E+00	9	2	5.59E+00	*
Offs-032	Parent-14	9	1	1.86E+00	Parent-18	9	1	3.04E+00	9	2	7.16E+00	*
Offs-033	Parent-13	9	2	-1.68E+00	Parent-42	9	2	-1.62E+00	9	3	8.50E-01	*
Offs-034	Parent-73	9	1	8.31E-01	Parent-78	9	2	-3.28E+00	9	3	2.27E-02	+
Offs-035	Parent-08	9	0	6.35E+00	Parent-14	9	1	-1.12E+00	9	3	1.51E+00	+
Offs-036	Parent-14	9	1	1.19E+00	Parent-18	9	1	3.78E+00	9	1	1.21E+01	*
Offs-037	Parent-14	9	1	2.87E+00	Parent-18	9	0	8.16E+00	9	2	9.85E+00	*
Offs-038	Parent-14	9	2	-1.33E+00	Parent-18	9	1	8.73E-01	9	3	1.78E+00	*
Offs-039	Parent-08	9	2	-2.82E+00	Parent-14	9	1	3.82E+00	9	3	3.08E+00	+
Offs-040	Parent-14	9	1	3.13E+00	Parent-73	9	1	-7.98E-01	9	2	7.01E+00	*
Offs-041	Parent-14	9	1	5.25E-02	Parent-18	9	2	-9.54E-01	9	2	5.27E+00	*
Offs-042	Parent-08	9	1	2.63E+00	Parent-14	9	0	7.30E+00	9	1	1.54E+01	*
Offs-043	Parent-08	9	1	6.70E-02	Parent-14	9	0	4.36E+00	9	1	9.17E+00	*
Offs-044	Parent-08	9	1	1.51E+00	Parent-14	9	0	5.36E+00	9	1	1.16E+01	*
Offs-045	Parent-02	9	1	3.31E+00	Parent-40	9	2	-1.52E+00	9	2	7.66E+00	*
Offs-046	Parent-14	9	0	7.82E+00	Parent-18	9	0	7.41E+00	9	1	1.54E+01	*
Offs-047	Parent-14	9	1	2.93E+00	Parent-18	9	1	1.64E+00	9	2	6.61E+00	*
Offs-048	Parent-14	9	1	2.20E+00	Parent-18	9	0	5.97E+00	9	2	6.33E+00	*
Offs-049	Parent-14	9	2	-6.27E-01	Parent-18	9	1	2.87E+00	9	2	9.31E+00	*
Offs-050	Parent-37	9	1	1.13E+01	Parent-79	9	2	-2.31E+00	9	3	1.11E+01	*
Offs-051	Parent-14	9	1	2.69E+00	Parent-18	9	0	6.56E+00	9	2	8.43E+00	*
Offs-052	Parent-14	9	0	8.19E+00	Parent-18	9	2	-2.37E+00	9	2	8.47E+00	*
Offs-053	Parent-14	9	1	1.74E+00	Parent-18	9	0	7.82E+00	9	1	1.20E+01	*

Offs-054	Parent-14	9	0	6.50E+00	Parent-18	9	0	9.27E+00	9	0	1.91E+01	*
Offs-055	Parent-18	9	1	2.85E+00	Parent-28	9	2	-4.55E+00	9	2	4.46E+00	*
Offs-056	Parent-18	9	0	9.56E+00	Parent-28	9	1	-1.29E+00	9	2	8.02E+00	*
Offs-057	Parent-14	9	0	7.48E+00	Parent-18	9	1	2.96E+00	9	2	9.47E+00	*
Offs-058	Parent-14	9	0	5.54E+00	Parent-18	9	1	2.67E+00	9	2	6.53E+00	+
Offs-059	Parent-14	9	2	-3.05E-01	Parent-18	9	0	6.90E+00	9	2	8.97E+00	*
Offs-060	Parent-15	9	2	-5.94E+00	Parent-73	9	0	6.72E+00	9	2	3.23E+00	*
Offs-061	Parent-18	9	1	2.75E+00	Parent-83	9	2	-1.34E+00	9	3	4.41E+00	*
Offs-062	Parent-08	9	2	-3.19E+00	Parent-14	9	0	9.33E+00	9	2	9.23E+00	*
Offs-063	Parent-22	9	1	5.31E+00	Parent-40	9	2	-2.58E-02	9	3	7.20E+00	*
Offs-064	Parent-14	9	0	9.06E+00	Parent-18	9	1	7.57E-01	9	2	9.20E+00	*
Offs-065	Parent-18	9	1	3.10E+00	Parent-28	9	3	-9.04E+00	9	3	2.02E-01	+
Offs-066	Parent-08	9	0	6.44E+00	Parent-14	9	0	8.82E+00	9	0	2.06E+01	*
Offs-067	Parent-14	9	1	2.19E+00	Parent-18	9	1	2.28E+00	9	2	7.87E+00	*
Offs-068	Parent-14	9	0	4.68E+00	Parent-18	9	0	8.84E+00	9	2	8.72E+00	*
Offs-069	Parent-14	9	0	6.37E+00	Parent-18	9	1	4.04E+00	9	2	9.07E+00	*
Offs-070	Parent-14	9	0	6.50E+00	Parent-18	9	0	1.05E+01	9	0	2.03E+01	*
Offs-071	Parent-14	9	0	8.08E+00	Parent-30	9	0	1.37E+01	9	0	2.52E+01	*
Offs-072	Parent-73	7	0	6.63E+00	Parent-89	7	1	-1.24E+00	7	1	5.96E+00	*
Offs-073	Parent-14	9	1	6.32E-01	Parent-73	9	0	4.69E+00	9	1	9.88E+00	*
Offs-074	Parent-14	9	1	2.00E+00	Parent-18	9	0	7.60E+00	9	1	1.25E+01	*
Offs-075	Parent-18	9	1	2.97E+00	Parent-28	9	2	-3.84E+00	9	3	1.08E+00	*
Offs-076	Parent-14	9	2	-1.04E+00	Parent-18	9	1	4.87E+00	9	3	6.03E+00	*
Offs-077	Parent-37	9	0	1.33E+01	Parent-79	9	2	-2.33E+00	9	2	1.36E+01	*
Offs-078	Parent-31	9	0	1.32E+01	Parent-85	9	1	6.72E+00	9	1	2.46E+01	*
Offs-079	Parent-14	9	1	1.52E+00	Parent-18	9	1	3.72E+00	9	2	6.59E+00	*
Offs-080	Parent-08	9	1	1.32E+00	Parent-14	9	1	3.70E+00	9	2	8.34E+00	*
Offs-081	Parent-14	9	0	8.94E+00	Parent-18	9	0	5.48E+00	9	1	1.40E+01	*

Offs-082	Parent-71	9	0	1.45E+01	Parent-76	9	1	2.08E+00	9	1	1.73E+01	*
Offs-083	Parent-08	9	1	1.15E+00	Parent-81	9	1	4.24E+00	9	2	8.97E+00	*
Offs-084	Parent-76	9	1	3.92E+00	Parent-81	9	0	8.58E+00	9	1	1.47E+01	*
Offs-085	Parent-08	9	1	1.36E-01	Parent-81	9	0	9.35E+00	9	1	1.34E+01	*
Offs-086	Parent-76	9	1	1.63E+00	Parent-81	9	2	-9.56E-01	9	2	6.10E+00	*
Offs-087	Parent-76	9	1	1.50E+00	Parent-81	9	1	7.42E-01	9	1	7.90E+00	*
Offs-088	Parent-08	9	1	4.59E+00	Parent-81	9	1	3.53E+00	9	2	9.80E+00	*
Offs-089	Parent-76	9	1	4.28E+00	Parent-81	9	1	2.24E+00	9	1	1.21E+01	*
Offs-090	Parent-08	9	0	8.77E+00	Parent-14	9	0	5.40E+00	9	0	1.95E+01	*
Offs-091	Parent-76	9	1	1.23E+00	Parent-81	9	1	6.55E+00	9	2	1.06E+01	*
Offs-092	Parent-33	9	2	-2.61E+00	Parent-81	9	1	5.73E+00	9	3	5.23E+00	*
Offs-093	Parent-08	9	1	5.75E-01	Parent-14	9	0	6.17E+00	9	1	1.12E+01	*
Offs-094	Parent-08	9	0	8.67E+00	Parent-14	9	0	7.76E+00	9	0	2.20E+01	*
Offs-095	Parent-76	9	1	2.94E+00	Parent-81	9	1	2.02E+00	9	2	7.07E+00	*
Offs-096	Parent-08	9	1	2.90E+00	Parent-14	9	0	7.62E+00	9	1	1.57E+01	*
Offs-097	Parent-08	9	1	1.45E+00	Parent-81	9	0	1.08E+01	9	1	1.52E+01	*
Offs-098	Parent-08	9	1	-2.21E-01	Parent-81	9	0	8.30E+00	9	1	1.23E+01	*
Offs-099	Parent-08	9	0	7.21E+00	Parent-14	9	0	5.72E+00	9	0	1.78E+01	*
Offs-100	Parent-08	9	0	8.58E+00	Parent-14	9	0	4.69E+00	9	0	1.86E+01	*
Offs-101	Parent-08	9	0	8.34E+00	Parent-14	9	0	5.63E+00	9	0	1.88E+01	*
Offs-102	Parent-08	9	1	4.47E+00	Parent-81	9	1	2.73E+00	9	2	8.96E+00	*
Offs-103	Parent-08	9	0	8.22E+00	Parent-14	9	0	7.89E+00	9	0	2.16E+01	*
Offs-104	Parent-08	9	0	9.18E+00	Parent-14	9	1	3.72E+00	9	1	1.72E+01	*
Offs-105	Parent-08	9	1	5.79E-01	Parent-14	9	0	5.80E+00	9	1	1.09E+01	*
Offs-106	Parent-76	9	1	1.45E+00	Parent-81	9	1	7.05E+00	9	2	1.06E+01	*
Offs-107	Parent-08	9	1	2.47E+00	Parent-81	9	0	8.34E+00	9	1	1.41E+01	*
Offs-108	Parent-76	9	0	5.25E+00	Parent-81	9	0	9.69E+00	9	1	1.59E+01	*
Offs-109	Parent-08	9	1	-5.44E-01	Parent-81	9	0	9.40E+00	9	1	1.29E+01	*

Offs-110	Parent-76	9	0	1.17E+01	Parent-81	9	0	7.31E+00	9	0	2.34E+01	*
Offs-111	Parent-08	9	0	8.53E+00	Parent-14	9	0	5.44E+00	9	0	1.93E+01	*
Offs-112	Parent-08	9	1	3.62E+00	Parent-14	9	0	7.39E+00	9	1	1.62E+01	*
Offs-113	Parent-08	9	1	2.32E+00	Parent-14	9	1	2.67E+00	9	2	8.92E+00	*
Offs-114	Parent-08	9	1	3.29E+00	Parent-14	9	0	5.58E+00	9	1	1.39E+01	*
Offs-115	Parent-76	9	2	1.44E+00	Parent-81	9	0	1.06E+01	9	2	1.38E+01	*
Offs-116	Parent-76	9	0	8.05E+00	Parent-81	9	1	1.94E+00	9	1	1.32E+01	*
Offs-117	Parent-76	9	0	7.14E+00	Parent-81	9	1	-1.02E-01	9	1	9.27E+00	*
Offs-118	Parent-08	9	0	8.58E+00	Parent-14	9	0	5.04E+00	9	0	1.90E+01	*
Offs-119	Parent-08	9	1	2.68E-01	Parent-81	9	1	5.92E+00	9	2	1.00E+01	*
Offs-120	Parent-08	9	1	1.72E+00	Parent-14	9	0	5.36E+00	9	1	1.21E+01	*
Offs-121	Parent-08	9	1	2.58E+00	Parent-81	9	0	4.44E+00	9	1	1.21E+01	*
Offs-122	Parent-76	9	0	1.17E+01	Parent-81	9	0	7.43E+00	9	0	2.36E+01	*
Offs-123	Parent-76	9	0	9.33E+00	Parent-81	9	0	7.08E+00	9	0	2.04E+01	*
Offs-124	Parent-08	9	1	1.90E+00	Parent-81	9	2	-2.88E+00	9	3	9.19E-01	*
Offs-125	Parent-08	9	0	4.72E+00	Parent-14	9	0	8.37E+00	9	0	1.84E+01	*
Offs-126	Parent-08	9	1	3.32E+00	Parent-81	9	1	5.79E+00	9	2	1.25E+01	*
Offs-127	Parent-08	9	0	5.80E+00	Parent-14	9	0	6.94E+00	9	0	1.76E+01	*
Offs-128	Parent-08	9	0	8.49E+00	Parent-14	9	0	8.39E+00	9	0	2.24E+01	*
Offs-129	Parent-08	9	1	2.97E-01	Parent-14	9	0	7.46E+00	9	1	1.27E+01	*
Offs-130	Parent-08	9	1	2.89E+00	Parent-14	9	0	4.72E+00	9	1	1.27E+01	*
Offs-131	Parent-08	9	1	1.35E+00	Parent-14	9	0	6.40E+00	9	1	1.29E+01	*
Offs-132	Parent-08	9	1	4.09E+00	Parent-81	9	2	-1.75E+00	9	3	5.48E+00	*
Offs-133	Parent-08	9	0	7.44E+00	Parent-14	9	0	5.40E+00	9	0	1.82E+01	*
Offs-134	Parent-08	9	0	8.17E+00	Parent-14	9	0	6.62E+00	9	0	2.03E+01	*
Offs-135	Parent-08	9	1	1.57E+00	Parent-14	9	0	7.44E+00	9	1	1.42E+01	*
Offs-136	Parent-08	9	1	2.87E+00	Parent-81	9	0	8.60E+00	9	1	1.50E+01	*
Offs-137	Parent-08	9	1	1.76E+00	Parent-14	9	0	4.36E+00	9	1	1.12E+01	*

Offs-138	Parent-76	9	0	8.77E+00	Parent-81	9	0	8.83E+00	9	0	2.04E+01	*
Offs-139	Parent-76	9	1	1.54E+00	Parent-81	9	2	-2.42E+00	9	2	4.94E+00	*
Offs-140	Parent-08	9	1	6.86E-01	Parent-81	9	0	8.52E+00	9	1	1.34E+01	*
Offs-141	Parent-08	9	1	2.02E+00	Parent-81	9	1	1.45E+00	9	2	4.92E+00	*
Offs-142	Parent-76	9	1	1.70E+00	Parent-81	9	2	-1.41E+00	9	2	6.41E+00	*
Offs-143	Parent-08	9	1	2.38E+00	Parent-14	9	0	3.71E+00	9	1	1.14E+01	*
Offs-144	Parent-67	9	1	4.15E+00	Parent-81	9	0	9.86E+00	9	1	1.48E+01	*
Offs-145	Parent-71	9	0	1.37E+01	Parent-76	9	0	1.18E+01	9	0	2.96E+01	*
Offs-146	Parent-08	9	1	4.84E-01	Parent-14	9	0	7.05E+00	9	1	1.25E+01	*
Offs-147	Parent-08	9	1	3.77E+00	Parent-81	9	0	1.13E+01	9	1	1.81E+01	*
Offs-148	Parent-08	9	0	7.16E+00	Parent-14	9	0	8.36E+00	9	0	2.08E+01	*
Offs-149	Parent-08	9	1	-5.53E-01	Parent-14	9	0	5.40E+00	9	1	9.33E+00	*
Offs-150	Parent-08	9	1	2.76E+00	Parent-81	9	1	1.94E+00	9	2	6.94E+00	*
Offs-151	Parent-08	9	1	1.25E+00	Parent-14	9	0	5.58E+00	9	1	1.13E+01	*
Offs-152	Parent-67	9	0	1.44E+01	Parent-76	9	0	1.10E+01	9	1	2.41E+01	*
Offs-153	Parent-08	9	1	3.51E+00	Parent-14	9	0	4.04E+00	9	1	1.26E+01	*
Offs-154	Parent-76	9	1	3.81E+00	Parent-81	9	0	9.79E+00	9	1	1.65E+01	*
Offs-155	Parent-08	9	0	8.81E+00	Parent-14	9	0	4.95E+00	9	0	1.91E+01	*
Offs-156	Parent-08	9	1	2.02E+00	Parent-81	9	1	9.53E-01	9	2	5.11E+00	*
Offs-157	Parent-08	9	1	2.10E+00	Parent-81	9	0	9.39E+00	9	1	1.58E+01	*
Offs-158	Parent-12	9	2	-1.39E+00	Parent-71	9	1	7.49E+00	9	2	1.15E+01	*
Offs-159	Parent-08	9	0	5.34E+00	Parent-14	9	0	7.68E+00	9	0	1.83E+01	*
Offs-160	Parent-76	9	1	3.54E+00	Parent-81	9	1	2.39E+00	9	2	8.87E+00	*
Offs-161	Parent-08	9	0	6.31E+00	Parent-14	9	1	2.66E+00	9	1	1.32E+01	*
Offs-162	Parent-76	9	0	7.92E+00	Parent-81	9	1	6.78E-01	9	1	1.11E+01	*
Offs-163	Parent-08	9	1	4.55E+00	Parent-81	9	2	-1.78E+00	9	3	4.80E+00	*
Offs-164	Parent-08	9	1	1.98E+00	Parent-14	9	0	5.49E+00	9	1	1.25E+01	*
Offs-165	Parent-08	9	1	3.54E+00	Parent-81	9	2	2.41E-01	9	3	3.75E+00	*

Offs-166	Parent-08	9	0	8.64E+00	Parent-14	9	0	7.80E+00	9	0	2.20E+01	*
Offs-167	Parent-76	9	1	4.99E-01	Parent-81	9	1	7.08E+00	9	2	9.78E+00	*
Offs-168	Parent-76	9	1	3.58E+00	Parent-81	9	2	-6.66E-01	9	2	8.76E+00	*
Offs-169	Parent-08	9	1	7.63E-01	Parent-14	9	0	7.54E+00	9	1	1.33E+01	*
Offs-170	Parent-76	9	1	4.25E+00	Parent-81	9	2	-1.51E+00	9	2	8.86E+00	*
Offs-171	Parent-08	9	1	2.86E+00	Parent-14	9	0	4.72E+00	9	1	1.26E+01	*
Offs-172	Parent-08	9	1	3.29E+00	Parent-81	9	1	1.95E+00	9	2	7.17E+00	*
Offs-173	Parent-08	9	0	4.45E+00	Parent-14	9	0	8.72E+00	9	0	1.85E+01	*
Offs-174	Parent-76	9	1	4.00E+00	Parent-81	9	1	1.18E+00	9	2	7.41E+00	*
Offs-175	Parent-08	9	2	-3.95E+00	Parent-14	9	0	7.46E+00	9	2	7.59E+00	*
Offs-176	Parent-08	9	0	4.50E+00	Parent-14	9	0	7.55E+00	9	0	1.73E+01	*
Offs-177	Parent-76	9	1	4.03E+00	Parent-81	9	1	1.66E+00	9	1	1.21E+01	*
Offs-178	Parent-76	9	1	1.69E+00	Parent-81	9	1	1.73E+00	9	1	9.63E+00	*
Offs-179	Parent-08	9	1	2.93E+00	Parent-81	9	1	3.30E+00	9	2	7.34E+00	*
Offs-180	Parent-08	9	1	-1.41E-01	Parent-14	9	0	6.03E+00	9	1	1.04E+01	*
Offs-181	Parent-08	9	0	5.34E+00	Parent-14	9	0	7.78E+00	9	0	1.84E+01	*
Offs-182	Parent-08	9	0	6.08E+00	Parent-14	9	0	5.49E+00	9	0	1.64E+01	*
Offs-183	Parent-08	9	1	2.88E+00	Parent-81	9	1	2.44E+00	9	2	6.72E+00	*
Offs-184	Parent-08	9	1	-5.69E-01	Parent-14	9	0	8.50E+00	9	1	1.29E+01	*
Offs-185	Parent-08	9	0	6.34E+00	Parent-14	9	0	6.82E+00	9	0	1.80E+01	*
Offs-186	Parent-08	9	1	-3.23E-01	Parent-14	9	0	6.95E+00	9	1	1.16E+01	*
Offs-187	Parent-08	9	1	-3.32E-01	Parent-81	9	0	9.05E+00	9	1	1.18E+01	*
Offs-188	Parent-08	9	1	2.03E+00	Parent-81	9	1	6.66E+00	9	2	1.20E+01	*
Offs-189	Parent-08	9	1	1.04E+00	Parent-14	9	0	5.12E+00	9	1	1.12E+01	*
Offs-190	Parent-08	9	0	7.72E+00	Parent-14	9	0	5.44E+00	9	0	1.85E+01	*
Offs-191	Parent-08	9	0	6.22E+00	Parent-14	9	0	8.82E+00	9	0	2.04E+01	*
Offs-192	Parent-08	9	0	4.63E+00	Parent-14	9	0	8.36E+00	9	0	1.83E+01	*
Offs-193	Parent-08	9	1	2.70E+00	Parent-81	9	1	7.75E-01	9	2	5.48E+00	*

Offs-194	Parent-08	9	1	7.63E-01	Parent-14	9	0	7.82E+00	9	1	1.36E+01	*
Offs-195	Parent-08	9	1	7.59E-02	Parent-14	9	0	5.53E+00	9	1	1.01E+01	*
Offs-196	Parent-08	9	0	8.27E+00	Parent-14	9	0	5.55E+00	9	0	1.92E+01	*
Offs-197	Parent-08	9	1	2.21E-01	Parent-81	9	1	6.01E-01	9	2	4.82E+00	*
Offs-198	Parent-76	9	1	4.14E+00	Parent-81	9	0	7.94E+00	9	1	1.49E+01	*
Offs-199	Parent-76	9	1	1.95E+00	Parent-81	9	2	-2.61E+00	9	2	4.75E+00	*
Offs-200	Parent-08	9	0	7.12E+00	Parent-14	9	0	6.23E+00	9	0	1.82E+01	*
Offs-183	Parent-08	9	1	2.88E+00	Parent-81	9	1	2.44E+00	9	2	6.72E+00	*
Offs-184	Parent-08	9	1	-5.69E-01	Parent-14	9	0	8.50E+00	9	1	1.29E+01	*
Offs-185	Parent-08	9	0	6.34E+00	Parent-14	9	0	6.82E+00	9	0	1.80E+01	*
Offs-186	Parent-08	9	1	-3.23E-01	Parent-14	9	0	6.95E+00	9	1	1.16E+01	*
Offs-187	Parent-08	9	1	-3.32E-01	Parent-81	9	0	9.05E+00	9	1	1.18E+01	*
Offs-188	Parent-08	9	1	2.03E+00	Parent-81	9	1	6.66E+00	9	2	1.20E+01	*
Offs-189	Parent-08	9	1	1.04E+00	Parent-14	9	0	5.12E+00	9	1	1.12E+01	*
Offs-190	Parent-08	9	0	7.72E+00	Parent-14	9	0	5.44E+00	9	0	1.85E+01	*
Offs-191	Parent-08	9	0	6.22E+00	Parent-14	9	0	8.82E+00	9	0	2.04E+01	*
Offs-192	Parent-08	9	0	4.63E+00	Parent-14	9	0	8.36E+00	9	0	1.83E+01	*
Offs-193	Parent-08	9	1	2.70E+00	Parent-81	9	1	7.75E-01	9	2	5.48E+00	*
Offs-194	Parent-08	9	1	7.63E-01	Parent-14	9	0	7.82E+00	9	1	1.36E+01	*
Offs-195	Parent-08	9	1	7.59E-02	Parent-14	9	0	5.53E+00	9	1	1.01E+01	*
Offs-196	Parent-08	9	0	8.27E+00	Parent-14	9	0	5.55E+00	9	0	1.92E+01	*
Offs-197	Parent-08	9	1	2.21E-01	Parent-81	9	1	6.01E-01	9	2	4.82E+00	*
Offs-198	Parent-76	9	1	4.14E+00	Parent-81	9	0	7.94E+00	9	1	1.49E+01	*
Offs-199	Parent-76	9	1	1.95E+00	Parent-81	9	2	-2.61E+00	9	2	4.75E+00	*
Offs-200	Parent-08	9	0	7.12E+00	Parent-14	9	0	6.23E+00	9	0	1.82E+01	*
Offs-201	Parent-76	9	0	1.26E+01	Parent-81	9	2	-3.61E+00	9	2	1.16E+01	*
Offs-202	Parent-08	9	1	3.49E+00	Parent-81	9	0	1.00E+01	9	1	1.65E+01	*
Offs-203	Parent-08	9	0	8.89E+00	Parent-14	9	0	7.44E+00	9	0	2.19E+01	*

Offs-204	Parent-08	9	1	2.76E+00	Parent-14	9	0	7.98E+00	9	1	1.59E+01	*
Offs-205	Parent-08	9	1	2.13E+00	Parent-14	9	0	3.68E+00	9	1	1.08E+01	*
Offs-206	Parent-76	9	0	8.68E+00	Parent-81	9	1	1.77E+00	9	1	1.33E+01	*
Offs-207	Parent-08	9	1	3.46E+00	Parent-81	9	1	-9.44E-01	9	2	5.84E+00	*
Offs-208	Parent-08	9	1	1.76E+00	Parent-14	9	0	4.99E+00	9	1	1.18E+01	*
Offs-209	Parent-08	9	1	1.44E+00	Parent-14	9	0	4.72E+00	9	1	1.06E+01	*
Offs-210	Parent-76	9	0	1.09E+01	Parent-81	9	1	3.41E+00	9	1	1.71E+01	*
Offs-211	Parent-76	9	0	1.14E+01	Parent-81	9	0	8.77E+00	9	0	2.42E+01	*
Offs-212	Parent-08	9	1	2.49E+00	Parent-14	9	0	7.61E+00	9	1	1.53E+01	*
Offs-213	Parent-76	9	1	7.65E+00	Parent-81	9	1	-1.47E+00	9	1	1.29E+01	*
Offs-214	Parent-08	9	1	1.20E+00	Parent-14	9	0	4.36E+00	9	1	1.00E+01	*
Offs-215	Parent-08	9	1	2.91E-01	Parent-14	9	0	6.49E+00	9	1	1.13E+01	*
Offs-216	Parent-71	9	1	6.30E+00	Parent-76	9	0	7.98E+00	9	2	1.22E+01	*
Offs-217	Parent-08	9	1	2.85E+00	Parent-81	9	1	3.91E+00	9	2	7.97E+00	*
Offs-218	Parent-08	9	0	9.02E+00	Parent-14	9	0	4.72E+00	9	0	1.91E+01	*
Offs-219	Parent-80	9	1	4.52E+00	Parent-81	9	0	8.31E+00	9	1	1.58E+01	*
Offs-220	Parent-08	9	1	1.16E+00	Parent-14	9	0	6.26E+00	9	1	1.19E+01	*
Offs-221	Parent-08	9	0	5.31E+00	Parent-14	9	0	6.08E+00	9	0	1.62E+01	*
Offs-222	Parent-80	9	1	3.16E+00	Parent-81	9	0	7.60E+00	9	2	1.08E+01	*
Offs-223	Parent-08	9	1	2.04E+00	Parent-81	9	0	6.82E+00	9	1	1.41E+01	*
Offs-224	Parent-76	9	2	-3.75E+00	Parent-81	9	0	1.07E+01	9	2	9.29E+00	*
Offs-225	Parent-08	9	0	6.09E+00	Parent-14	9	0	5.85E+00	9	0	1.68E+01	*
Offs-226	Parent-08	9	1	3.09E-01	Parent-81	9	0	7.43E+00	9	1	1.22E+01	*
Offs-227	Parent-08	9	0	5.80E+00	Parent-14	9	0	5.77E+00	9	0	1.64E+01	*
Offs-228	Parent-08	9	1	1.80E+00	Parent-81	9	1	1.06E+00	9	2	5.00E+00	*
Offs-229	Parent-08	9	1	7.57E-01	Parent-14	9	0	4.72E+00	9	1	9.96E+00	*
Offs-230	Parent-08	9	0	6.99E+00	Parent-14	9	0	5.85E+00	9	0	1.77E+01	*

* indicated > 0.95, and + indicated > 0.80
